# Rapid and easy-to-use ES cell manipulation device with a small groove near culturing wells

**DOI:** 10.1186/s13104-020-05294-w

**Published:** 2020-10-05

**Authors:** Shun-ichi Funano, Daisuke Tone, Hideki Ukai, Hiroki R. Ueda, Yo Tanaka

**Affiliations:** 1 Laboratory for Integrated Biodevice, Center for Biosystems Dynamics Research, RIKEN, 1-3 Yamadaoka, Suita, Osaka, 565-0871 Japan; 2 Laboratory for Synthetic Biology, Center for Biosystems Dynamics Research, RIKEN, 1-3 Yamadaoka, Suita, Osaka, 565-0871 Japan; 3 International Research Center for Neurointelligence (WPI-IRCN), UTIAS, The University of Tokyo, 7-3-1 Hongo, Bunkyo, Tokyo, 113-0033 Japan; 4 Department of Systems Pharmacology, Graduate School of Medicine, The University of Tokyo, 7-3-1 Hongo, Bunkyo, Tokyo, 113-0033 Japan

**Keywords:** Colony, ES cell, Genome editing, Rapid manipulation

## Abstract

**Objective:**

Production of genetically modified mice including Knock-out (KO) or Knock-in (KI) mice is necessary for organism-level phenotype analysis. Embryonic stem cell (ESC)-based technologies can produce many genetically modified mice with less time without crossing. However, a complicated manual operation is required to increase the number of ESC colonies. Here, the objective of this study was to design and demonstrate a new device to easily find colonies and carry them to microwells.

**Results:**

We developed a polydimethylsiloxane-based device for easy manipulation and isolation of ESC colonies. By introducing ESC colonies into the groove placed near culturing microwells, users can easily find, pick up and carry ESC colonies to microwells. By hydrophilic treatment using bovine serum albumin, 2-μL droplets including colonies reached the microwell bottom. Operation time using this device was shortened for both beginners (2.3-fold) and experts (1.5-fold) compared to the conventional colony picking operation. Isolated ESC colonies were confirmed to have maintained pluripotency. This device is expected to promote research by shortening the isolation procedure for ESC colonies or other large cells (e.g. eggs or embryos) and shortening training time for beginners as a simple sorter.

## Introduction

Genetically modified mice including Knock-out (KO) or Knock-in (KI) mice are necessary for organism-level phenotype analysis. To analyze organism-level phenotype formed from complex molecular/cellular networks, preparing many types of genetically modified mice is necessary. However, their production requires tremendous time and space. Development of simple and easy methods are desirable.

One reason for low-throughput is complicated crossing process taking several months/year. Repeated backcrosses are needed to obtain complete KO/KI homozygotes. To allow completion of KO/KI mouse production and phenotyping analysis within F0 generation, next-generation mammalian genetics without crossing was proposed [1, 2]. The process introduces designed mutation into the genomic DNA of mammalian zygotes or embryonic stem cells (ESCs) using site-specific endonucleases including a clustered regularly interspaced short palindromic repeat (CRISPR)/CRISPR-associated protein 9 system [3]. Introduction of triple-target CRISPR into zygotes efficiently generates whole-body bi-allelic KO mice with almost 100% probability [4]. However, KI efficiency is still not high enough without crossing [5]. F0 mice obtained by KI into zygotes are often mosaic. By contrast, genome-editing methods were also applied to ESCs to generate KI ESCs with high efficiency [6]. Moreover, 3i-medium-cultivated KI ESCs injected into 8-cell stage embryos or aggregated with 8-cell stage embryonic cells can generate completely ESC-derived KI mice [6–8]. Thus, ESC-based methods produce KI mice without crossing.

However, ESC-based methods still have issues. Many colonies are necessary to be analyzed genetically, which needs colony picking operation requiring special skills: first, finding colonies on the dish bottom and, second, bringing single colonies carefully to each microwell. Reducing complicated manual handling is important. Recently, we established a protocol manipulating ESC colonies using a conventional cell-sorting machine [6]. However, it is expensive, large, and difficult be introduced into all biological laboratories. “User-friendly” concept is necessary such as what we demonstrated for cell patterning [9, 10] or manipulation [8, 11].

**Fig. 1:**
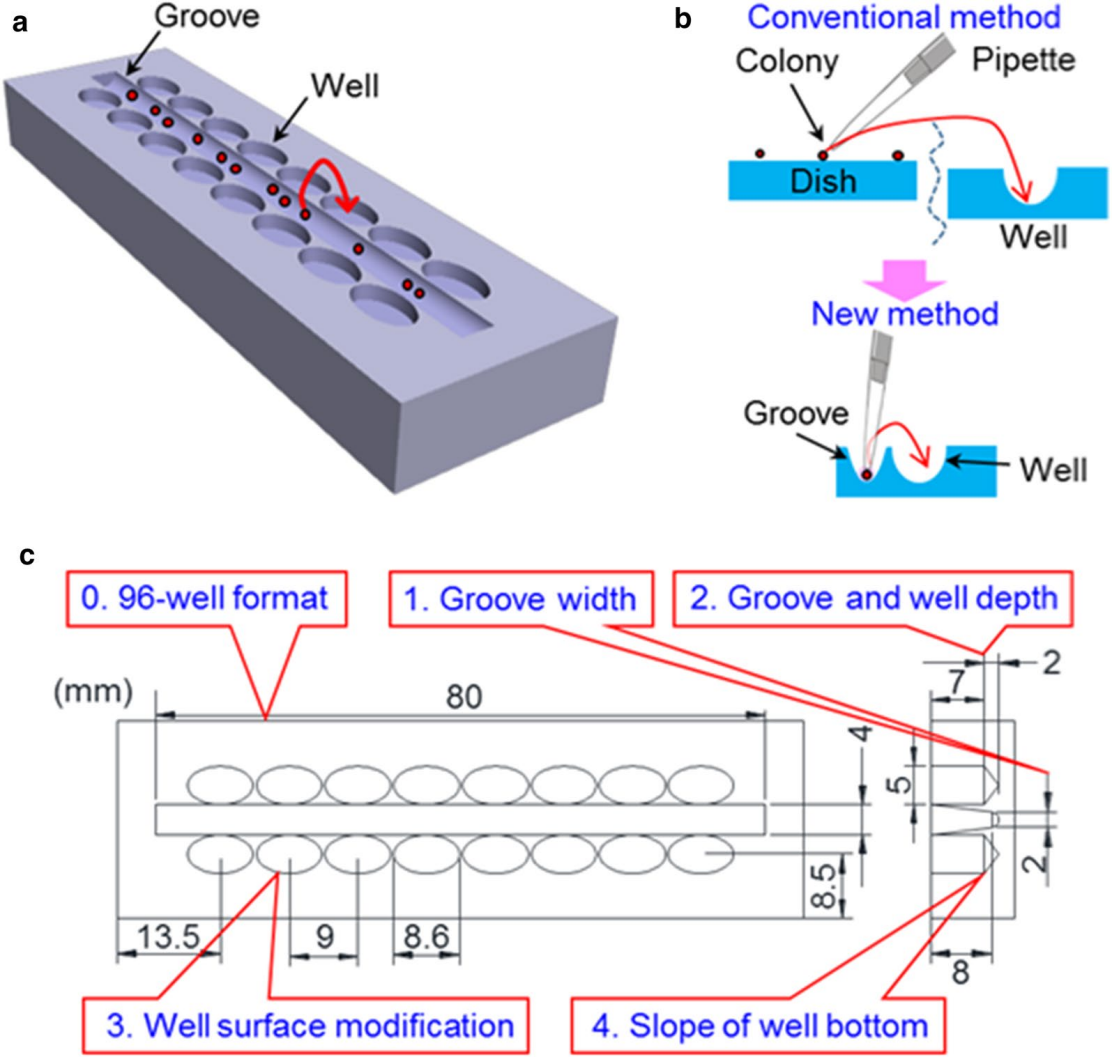
Concept of the device. **a** 3D view of the device with a groove and microwells near it. **b** Illustration comparing the method using the device compared with the conventional method. **c** Design of the device indicating the dimensions

Here, we designed a new device to easily find colonies and carry them to microwells (Fig. 1). By a groove in the device center, introduced colonies are aligned in the groove bottom, which can be easily found. Also, microwells are placed near the groove to easily carry cells from the groove to wells. In this study, we fabricated the device and demonstrated faster operation time compared with conventional colony picking.

## Main text

### Methods

More detailed procedures and information of procurement are described in Additional file 1.

#### Device fabrication

Polydimethylsiloxane (PDMS) was used due to its biocompatibility and easily molded property [12]. Based on design data prepared with computer-aided design software, an aluminum mold was made by a metalworking company. PDMS pre-polymer and a curing agent were mixed (10:1), poured into the mold, cured at 70 °C for 3 h and peeled off.

#### Surface treatment

For fluorosilane treatment, each well was filled with fluorine coating reagent containing poly-fluorosilane. It was promptly discarded, and the device was dried at 70 °C for 1 h. For bovine serum albumin (BSA) treatment, each well was filled with 1% BSA water solution and removed after 30 min.

#### Bead sliding down measurement

Four kinds of heights (1, 2, 3, and 4 mm) at 4-mm distant point from support contact position of a glass plate were set. 2-μL suspension (50 beads/μL) of 45-μm beads was placed on the point. After 30 s, the plate was positioned horizontally and air-dried. Bead number at each position was counted using a microscope.

#### Preparation and isolation of ESC colonies

ESC colonies were prepared as follows [6]. C57BL/6 (B6) ESCs were seeded (1 × 10^5^ cells) in amine-coated 6-well plates, and cultivated at 37 °C in 5% CO_2_ under humidified conditions with 3i culture medium. After 3 days, ESC colonies were isolated. In conventional method, a single colony selected under the microscope was sucked with a pipette and transferred to a 96-well plate. When using the ESC manipulation device, colonies were detached from a plate by gentle pipetting and dispersed in the device groove. A single colony in the groove was selected under the microscope, sucked up with a pipette, and directly dispensed into a well of the device.

#### Production of ESC-derived mice (ES-mice)

ES-mice were produced as described previously [6]. B6 ESC single colony separated from the ESC manipulation device was expanded and cultivated in an amine-coated 6-well plate. The expanded colonies were trypsinized and dissociated into single cells. 10–30 ESCs were injected into each ICR 8-cell-stage embryo, and the embryos were transferred into the uteri of a pseudopregnant ICR female mouse. Contribution of ESCs in a chimeric mouse was determined by its coat color. All the animals used to produce the results shown in this article were sedated with carbon dioxide, then euthanized by cervical dislocation.

### Results

#### Results of device fabrication and groove width optimization

The well shape, groove width and depth were considered for designing the device. The well bottom was designed as a cone so that the ESC colony would slide down to the center of this bottom. The well spacing of the device was adjusted to the well spacing of a 96-well plate so that multichannel pipettes could be used. In addition, the well was designed to be elliptical to have sufficient volume for injecting post-processing solutions.

Three devices with a groove width (1, 2, and 4 mm) were manufactured. Colonies were dispersed in each groove and observed under the microscope. When the groove width was 2 mm, colonies were arranged in a line at the groove bottom. When the width was 4 mm, colonies were scattered at the bottom (Additional file 2: Fig. S1a). When the width was 1 mm, transferring colonies from the groove to well was difficult. Therefore, optimum width was 2 mm. The groove depth was designed to be same as the well depth to observe both bottoms of the groove and well in same field of view under a microscope (Additional file 2: Fig. S1b). The aluminum mold was made accordingly, and the device was made as designed (Additional file 2: Fig. S1c).

#### Validation of surface treatment

**Fig. 2:**
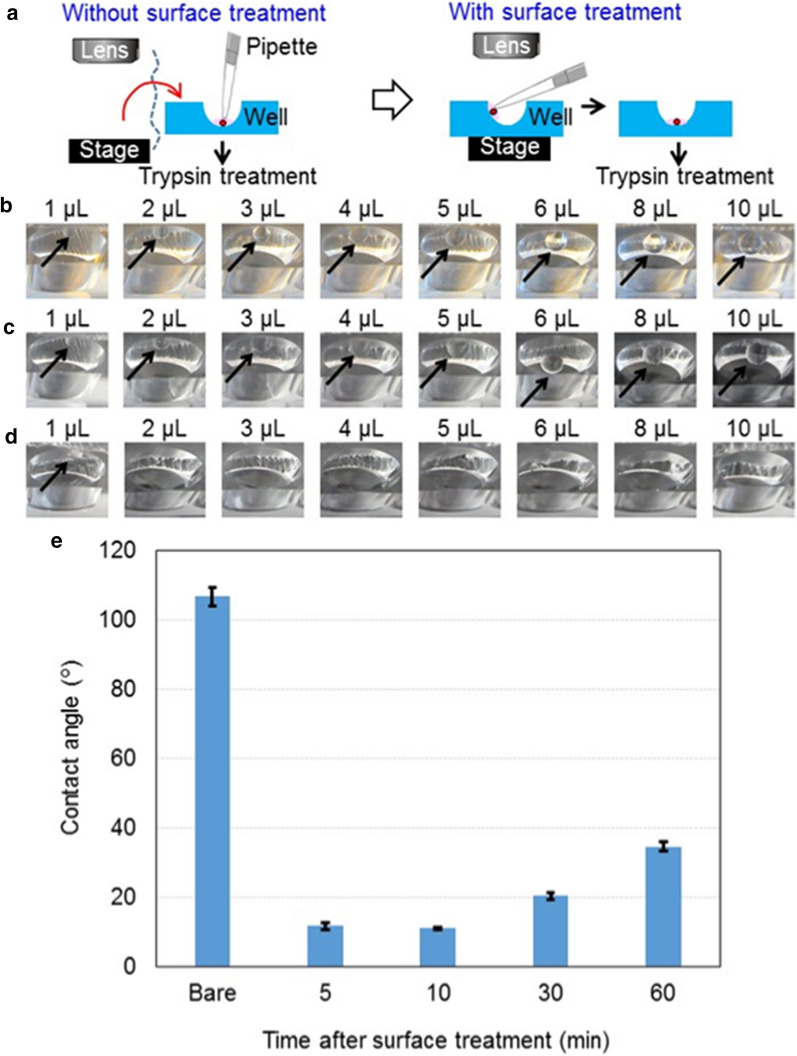
| Investigation of well surface treatment. **a** Illustration related to modifying the well surface to disperse droplets to the well bottom. **b-d** Photos showing the well with a droplet on the wall for three kinds of treated surfaces: (b) bare polydimethylsiloxane (PDMS), **c** fluorosilane-treated, and **d** 1% bovine serum albumin (BSA)-treated. **e** Time dependency of contact angles of 1% bovine serum albumin (BSA)-treated polydimethylsiloxane (PDMS) surfaces with control data for a bare PDMS surface

When transferring colonies from the groove to well during observation under the microscope, dispensing the droplet directly on the well bottom is difficult because the pipette hits the microscope lens. However, dispensing the droplet after moving ESC colony manipulation device from the microscope stage takes time. These problems can be solved by tilting the pipette and letting the droplet slide down the wall surface of the well to the bottom by surface treatment effect. An ESC colony contained drop contacted into the well wall can be slid down to the bottom quickly. Therefore, further time reduction is expected (Fig. 2a).

Medium droplets (1-10 μL, 1-μL steps) were dispensed on the well walls of untreated, fluorosilane-treated, and BSA-treated devices (Fig. 2b–d), indicating that when 2 μL or more volumes were dispensed, they slid down the BSA-treated wall to the bottom easily.

#### Investigation of duration of BSA treatment effect

Duration of BSA treatment effect was measured. PDMS sheets were immersed in 1% BSA solution, and blown off with an air-gun after 30 min. After 5, 10, 30, and 60 min, water contact angle was measured with a contact angle meter (DMs-401, Kyowa Interface Science, Japan) (Fig. 2e). The contact angle gradually increased with time after BSA treatment, but similar within 10 min, indicating that BSA treatment should be just before use.

#### Measurement of the bead sliding down effect due to the well wall slope

Although droplets slid down to the well bottom when the surface was BSA-treated, a colony contained in droplet might remain on the well wall surface. Therefore, we examined how beads actually slid down in various slope angles (Additional file 3: Fig. S2a). Beads clearly slid down when the slope angle was 45° or more (Additional file 3: Fig. S2c). Therefore, the colony would slide down to the bottom by dispensing the droplet on the well wall surface in the minor axis direction because the slope near the well bottom was 45° (Additional file 3: Fig. S2b). As Fig. S2d (Additional file 3), the colony slid down to the center of the bottom when culture medium containing the colony was dispensed.

#### Demonstration of shortened operation time using the device

**Fig. 3:**
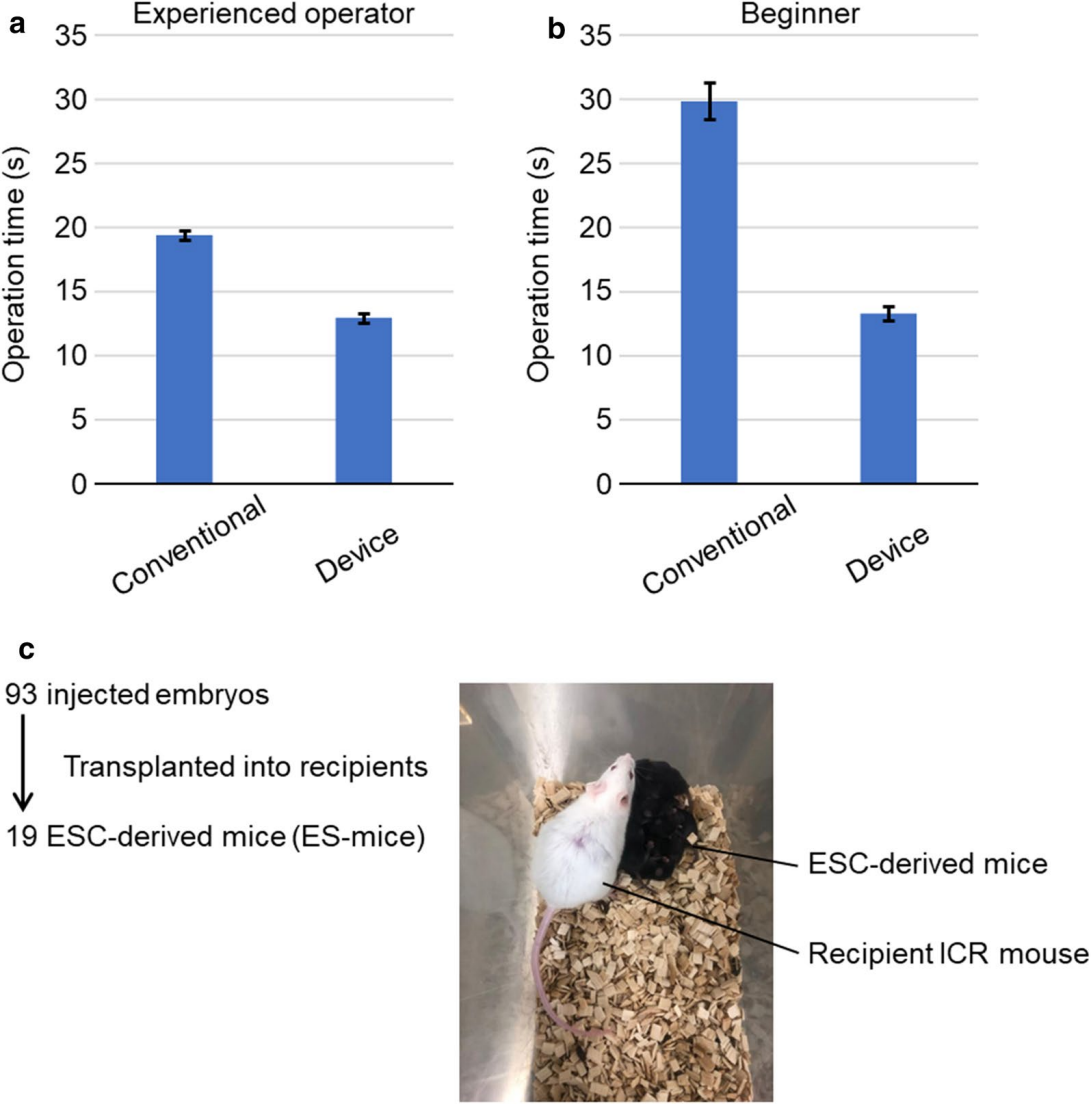
ESC manipulation device improves user operating time. **a, b** The operation time to pick up an ESC colony is indicated. Values are represented as mean and standard error; *n* = 4. **a** Results obtained by an experienced operator. **b** Results operated by a beginner. **c** A photo of ESC-derived mice (black ones) with recipient ICR mouse (white one)

An experienced operator (over 5 years’ experience) and a beginner (less than 1 years’ experience) transferred colonies 4 times each by conventional method and the device. Required time was measured (Fig. 3a, b). Actual operations by a beginner can be compared in Movie S1 (Additional file 4) (conventional method) and Movie S2 (Additional file 5) (using the device). The operation time became short by using the device for both the beginner (2.3-fold) and expert (1.5-fold). Especially for the beginner, time reduction was remarkable, and the beginner achieved operation time comparable to that of an expert.

#### Confirmation of producibility of ESC-derived mice (ES-mice)

In our method, ESC colonies were detached from a culture dish for single colony isolation. In such a situation, colonies might contact with each other and cross-contaminate in suspension. To validate the possibility of colony-to-colony cross-contamination, ESC colonies expressing reporter genes (EGFP or mKate2) were prepared and mixed into a suspension (Additional file 6: Figure S3a), and then isolated to single colonies with the ESC manipulation device. In the isolated 16 colonies, only one reporter gene, either EGFP or mKate, was expressed, and no double-positive colonies were detected (Additional file 6: Figure S3b). This result suggested that the cross-contamination in our method is a rare event, if any.

To examine the effect of the ESC colony manipulation device on the pluripotency of ESCs, the contribution of the isolated ESCs to chimeric mice was tested. The separated ESCs were injected into 8-cell embryos and transplanted into recipient ICR mice. As a result of transferring 93 early embryos injected with ESCs into 3 recipient ICR mice, we obtained 19 chimeric mice, and all of them are 100% ESC-contributed mice (ES-mice) (Fig. 3c). This result indicated that ESCs manipulated with our device maintained pluripotency.

### Discussion

Simple and high-throughput cell-sorting systems were reported using microfluidic chips recently [13–15]. They separate cells without special detecting/sorting devices, however, they only treat small particles/cells under 20 μm due to the limit of microfluidic size capability. Generally, they cannot be applied for ESC colonies larger than 100 μm. Although droplet sorters [16, 17] sort relatively large size droplets, still cannot handle large colonies. Several 3D culture systems on-chips handling and analyzing colonies/spheroids were also reported [18–21], but cannot separate colonies.

Recently, we established a protocol manipulating ESC colonies using a state-of-the-art cell-sorting machine (without microfluidics), which is adaptable to large cells/colonies [6]. However, usually, cell-sorting machines are integrated systems of microfluidics, optical detection and information processing [22–24]. Such systems may be expensive/large, which are difficult to be introduced into biological laboratories. In contrast, our newly developed device can be introduced everywhere due to its low-cost and small installation space requirement. Our device requires no special skills/equipment, and can be utilized even by inexperienced persons. In addition, it can be applied flexibly for various types of large cells (e.g. eggs/embryos) without any readjustment, enabling quick, parallel sample preparation in developmental and cell biology experiments, including whole egg/embryo imaging or gene expression analyses.

Automation of biological experiments is a current topic of debate with respect to reproducibility and being labor-intensive processes [25, 26]. In mice production, an isolated colony must be separated into single cells and dispensed to a culture dish. These processes require much time and effort. Our new device was designed with 96-well plate format to be compatible with multichannel pipettes. In future, these downstream processes (separating, dispensing, and culturing) could be automated using commercially available liquid handling platforms including Biomek and epMotion. Such an approach reduces labor force and realize high-throughput mammalian genetics offering comprehensive understanding of gene functions involving various organism-level phenomena.

The developed device can be developed into a portable device that is combined with other on-chip devices. Together with other on-chip, miniaturized microfluidic devices including miniaturized pumps [27, 28] or valves [29–31], full automated colony picking operations without manual handling can be realized in future.

## Limitations

This device can be alternative to cell-sorting machines, but generally applied to larger objects over 50 μm.

## Supplementary information

**Additional file 1:** Supplemental materials and methods.

**Additional file 2:** Supplemental Fig. S1.

**Additional file 3:** Supplemental Fig. S2.

**Additional file 4:** Supplemental Movie S1.

**Additional file 5:** Supplemental Movie S2.

**Additional file 6:** Supplemental Fig. S3.

## Data Availability

All data generated or analysed during this study are included in this published article and its supplementary information files.

## Abbreviations

KO: Knock-out
KI: Knock-in
ESC: Embryonic stem cell
CRISPR: Clustered regularly interspaced short palindromic repeat
PDMS: Polydimethylsiloxane
BSA: Bovine serum albumin
PBS: Phosphate-buffered saline
ES-mice: ESC-derived mice
