# Supplementary material for: Rapid and easy-to-use ES cell manipulation device with a small groove near culturing wells
Source: BMC Res Notes. 2020 Oct 5;13:453. doi: 10.1186/s13104-020-05294-w (PMC7534166; doi:10.1186/s13104-020-05294-w)
Supplement: Supplementary file 1 — Additional file 1: Supplemental materials and methods. [file 13104_2020_5294_MOESM1_ESM.docx]

Additional file 1

Rapid and easy-to-use ES cell manipulation device with a small groove near culturing wells

Shun-ichi Funano, Daisuke Tone, Hideki Ukai, Hiroki R Ueda & Yo Tanaka

### Materials and methods

### Device fabrication

### For the investigations we prepared ESC manipulation devices of one basic design made of polydimethylsiloxane (PDMS) (SILPOT 184, DuPont Toray Specialty Materials, Tokyo, Japan) in the following manner. PDMS was used because it is biocompatible and easily molded to make a prototype [12]. Briefly, the design data of the aluminum mold used in PDMS molding were prepared with computer-aided design (CAD) software (AutoCAD LT 2014, Autodesk, CA, USA), and an aluminum mold based on the CAD data was made by a general metalworking company (Ono Denki, Tokyo, Japan). The PDMS pre-polymer and a curing agent were mixed at a ratio of 10 to 1. The PDMS mixture was poured into the aluminum mold and cured at 70°C for 3 h. After that, the cured PDMS elastomer was removed from the aluminum mold.

**Surface treatment method**

In the case of the fluorosilane treatment, each well of the ESC manipulation device was filled with fluorine coating reagent containing poly fluorosilane (Novec 1720, 3M, MN, USA). After filling, the fluorine coating reagent was promptly discarded, and the ESC manipulation device was dried at 70°C for 1 h. In the case of the bovine serum albumin (BSA) treatment, each well of the ESC manipulation device was filled with 1% BSA water solution. After 30 min, the BSA solution was removed.

**Bead sliding down measurement**

A glass plate (C024401, Matsunami Glass Ind., Osaka, Japan) was set as follows: four heights of 1, 2, 3, and 4 mm were marked for a position 4 mm horizontally from the support contact position of the glass plate. 2 μL of a 50 beads/μL suspension of 45-μm diameter beads (Fluoresbrite (18242), Polysciences, PA, USA) was placed on the marked height position. After 30 s, the glass plate was positioned horizontally and air-dried. Then, bead positions were observed with a microscope (VHX-1000, KEYENCE, Osaka, Japan) equipped with a zoom lens (VH-Z100R), and the number of beads at each position was counted.

**Preparation and isolation of ESC colonies**

ESC colonies were prepared according to the following reported procedure [6]. C57BL/6 (B6) ESCs were seeded at 1 × 10^5^ cells in each well of an amine-coated 6-well plate (356721, Becton Dickinson Biosciences, NJ, USA), and the cultivation was started at 37°C in 5% CO_2_ under humidified conditions with 3i culture medium (Y40010, Takara Bio, Shiga, Japan). After 3 days, the ESC colonies were isolated as follows.

In the case of the conventional method, the culture plate with ESC colonies was placed on a microscope stage (M125, Leica Microsystems, Hesse, Germany). A single colony, selected under the microscope, was sucked up with a pipette. The culture plate was removed from the stage, a new 96-well plate (NJ111, Takara Bio, Shiga, Japan) containing 2 µL of PBS solution was placed on the stage, and the colony and culture medium in the pipette were dispensed into a well of the plate. Finally, the colony was observed with the microscope and found to have settled to the bottom of the well.

When using the ESC manipulation device, each well of the device was treated with 1% BSA solution in advance of the transfer operation. The BSA solution was removed and replaced with 2 μL of PBS solution just before use. ESC colonies were detached from the plate by gentle pipetting with a 1 mL pipette-tip and dispersed in the groove of the device. A single colony in the groove was selected under the microscope (M125, Leica Microsystems, Hesse, Germany), sucked up with a pipette, and the directly dispensed into a well of the device. Then, the microscope observation confirmed that the colony had settled to the bottom of the well.

For cross-contamination check, Rosa26::H2B-EGFP (EGFP) and Rosa26::NLS-mKate2 (mKate2) C57BL/6 ESCs were cultured on an amine-coated 6-well plate (356721, Becton Dickinson Biosciences, NJ, USA) at 37°C in 5% CO_2_ under humidified conditions with 3i culture medium (Y40010, Takara Bio, Shiga, Japan). The EGFP and mKate2 ESC colonies were detached respectively from the plate by gentle pipetting with a 1 mL pipette-tip and mixed in a 50 mL conical tube (227261, greiner bio-one). The mixed ESC colonies were left at room temperature for 5 min, allowing colonies contact with each other as in the actual operation of preparing colony suspension and then transferred to a 35 mm petri dish (351008, Falcon) for observation. Fluorescence images of the colony suspension were acquired with BX51 microscope (Olympus, Tokyo, Japan). Single colonies were isolated with the ESC manipulation device from the colony suspension and then transferred to each well of a 96-well clear bottom plate (353948, Falcon) with 3i culture medium for observation. Fluorescence images of isolated single colony in 96-well plate were acquired with IX71 microscope (Olympus, Tokyo, Japan).

**Production of ESC-derived mice (ES-mice)**

An amine-coated 6-well plate (356721, Becton Dickinson Biosciences, NJ, USA) were incubated at 37°C for overnight with 1% gelatin water solution (G1393, Sigma-Aldrich) in advance of cell culture. The B6 ESCs were seeded at 1 × 10^5^ cells and cultivated in the gelatin-coated 6-well plate at 37°C in 5% CO_2_ under humidified conditions with 3i culture medium (Y40010, Takara Bio, Shiga, Japan). For single colony isolation, add 100 μL of 2 mg/mL collagenase(CLS-3, Worthington Biochemical) to the culture medium in the plate and incubate 37°C in 5% CO_2_. Floated ESC colonies are collected and transferred to the ESC manipulation device, and single ESC colonies were isolated to each well of the device. 50 μL of 0.25% trypsin-EDTA solution (15090-046, Gibco) was added to the each well of the device containing ESC colonies, and they were incubated for 3 min at room temperature. 150 μL of mES medium was added to each well, and single-cell suspensions were made by gently pipetting the medium up and down several times and transferred to an amine-coated 24-well plate (356723, Becton Dickinson Biosciences, NJ, USA) to be expanded.

For ESCs injection into embryos, single-colony derived ESCs cultured in an amine-coated 6-well plate were treated with 300 μL of 0.25% trypsin-EDTA (15090-046, Gibco) for 1 min at room temperature and then dissociated into single cells by gentle pipetting. 10-30 ESCs were injected into an 8-cell-stage ICR embryo and cultured in KSOM-medium drops (KSOMaa, ARK Resource) covered with mineral oil until the next day. The embryos were transferred into the uteri of 2.5-dpc pseudopregnant ICR female mice. The contribution of the ESCs in an obtained chimeric mouse was determined by its coat color.
