# Supplementary material for: Rapid and easy-to-use ES cell manipulation device with a small groove near culturing wells
Source: BMC Res Notes. 2020 Oct 5;13:453. doi: 10.1186/s13104-020-05294-w (PMC7534166; doi:10.1186/s13104-020-05294-w)
Supplement: Supplementary file 2 — Additional file 2: Supplemental Fig. S1. [file 13104_2020_5294_MOESM2_ESM.pdf]

Additional file 2: Fig. S1

Rapid and easy-to-use ES cell manipulation device with a small groove near culturing wells

Shun-ichi Funano, Daisuke Tone, Hideki Ukai, Hiroki R Ueda & Yo Tanaka

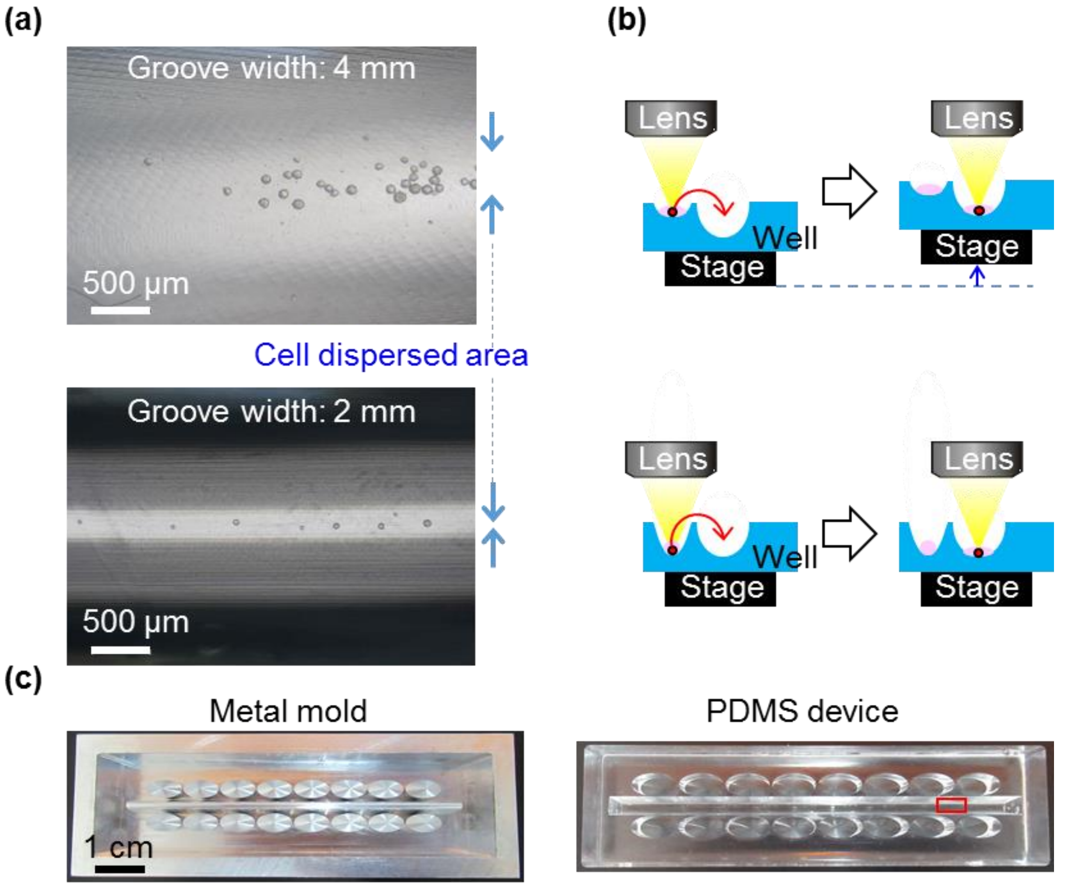

Fig. S1 Investigations of the groove width and the depth of both the groove and wells. (a) Microscope images related to the groove width showing embryonic stem cell (ESC) colonies dispersed in the grooves of 2- and 4-mm widths. (b) Illustration related to the depth of both the groove and wells, making the groove and well the same depth for simple operation. If they are different, the height of the stage must be adjusted every time. (c) Photos of a metal mold (left) and a polydimethylsiloxane (PDMS) device (right) for ESC colony manipulation.
