# Supplementary material for: Rapid and easy-to-use ES cell manipulation device with a small groove near culturing wells
Source: BMC Res Notes. 2020 Oct 5;13:453. doi: 10.1186/s13104-020-05294-w (PMC7534166; doi:10.1186/s13104-020-05294-w)
Supplement: Supplementary file 3 — Additional file 3: Supplemental Fig. S2. [file 13104_2020_5294_MOESM3_ESM.pdf]

Additional file 3: Fig. S2

Rapid and easy-to-use ES cell manipulation device with a small groove near culturing wells

Shun-ichi Funano, Daisuke Tone, Hideki Ukai, Hiroki R Ueda & Yo Tanaka

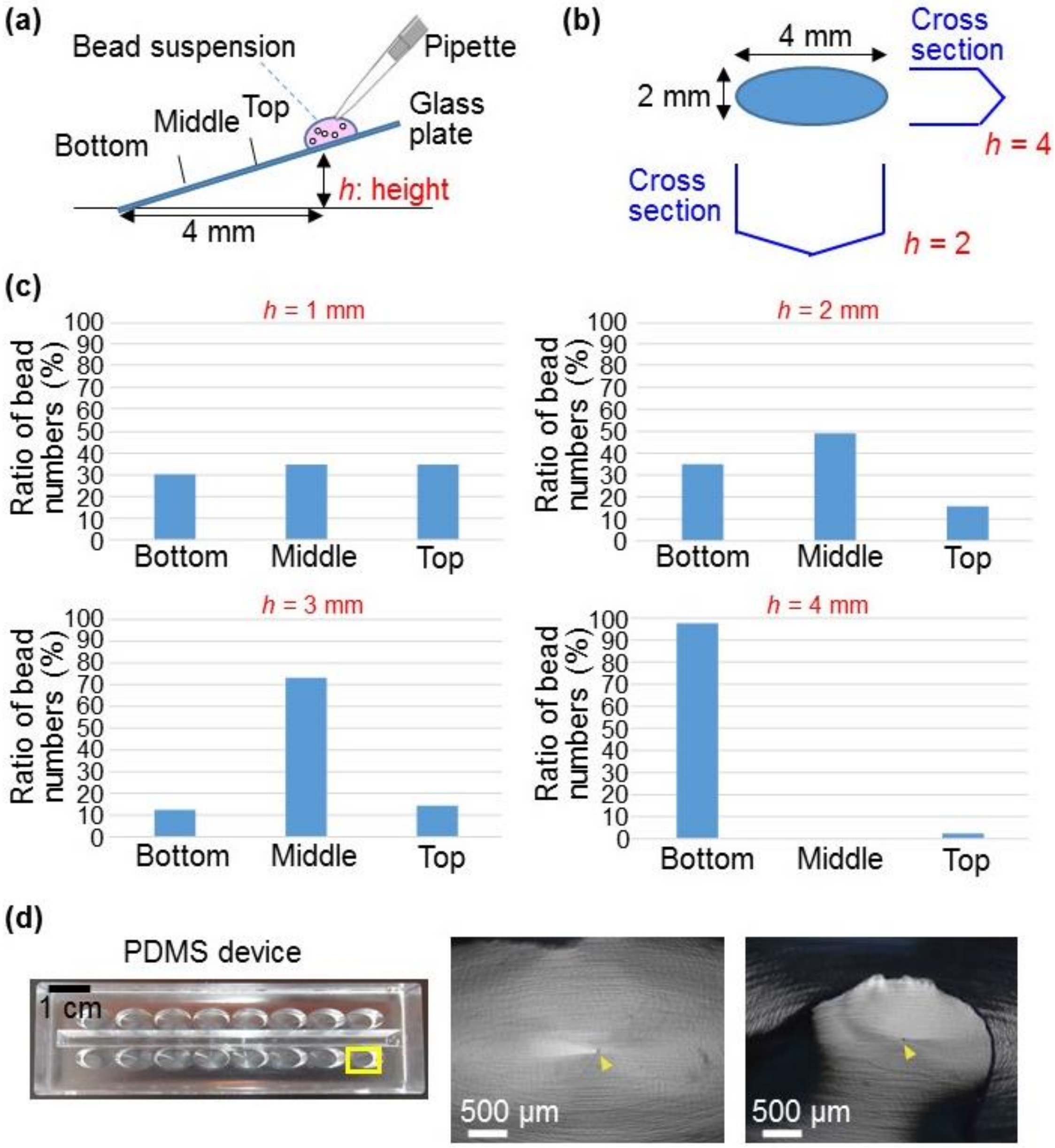

Fig. S2 Investigation of the slope of the well bottom. (a) Illustration for the slope of the well bottom, using various glass plate slopes to set the experimental condition for bead counting. (b) Actual well bottom shape on the device. (c) Experimental data showing the ratio of residual beads on the top, middle and bottom of the slopes at 4 conditions (*h* = 1, 2, 3, and 4 mm). (d) Photos showing the observed part in the device (left, yellow rectangle), and embryonic stem cell (ESC) colonies on the bottom of the wells in 4-µL droplets (middle and right).
