# Supplementary material for: Rapid and easy-to-use ES cell manipulation device with a small groove near culturing wells
Source: BMC Res Notes. 2020 Oct 5;13:453. doi: 10.1186/s13104-020-05294-w (PMC7534166; doi:10.1186/s13104-020-05294-w)
Supplement: Supplementary file 6 — Additional file 6: Supplemental Fig. S3. [file 13104_2020_5294_MOESM6_ESM.pdf]

Additional file 6: Fig. S3

Rapid and easy-to-use ES cell manipulation device with a small groove near culturing wells

Shun-ichi Funano, Daisuke Tone, Hideki Ukai, Hiroki R Ueda & Yo Tanaka

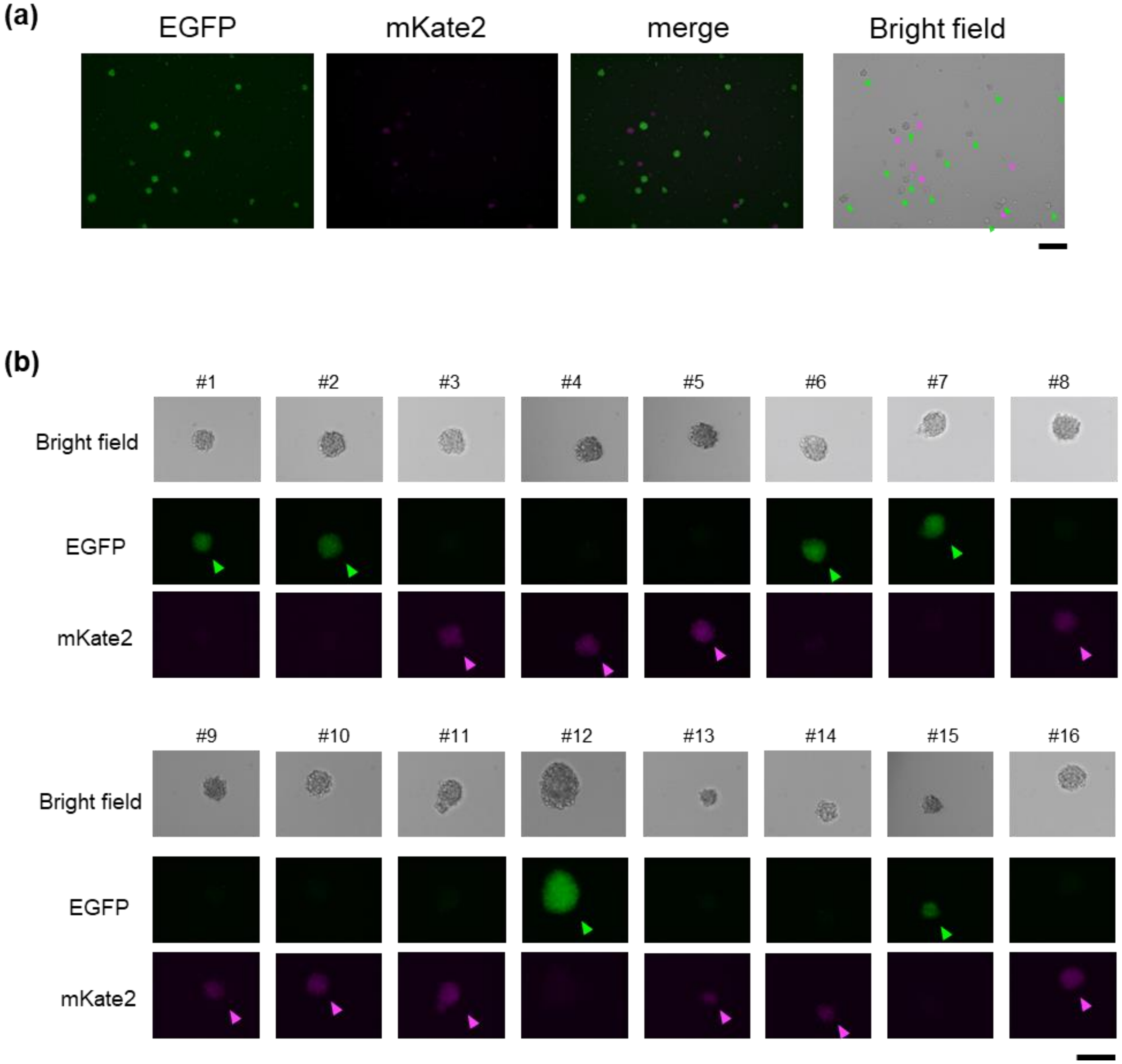

Fig. S3 Cross-contamination-free single colony isolation with the ESC manipulation device. (a) Representative bright field and fluorescence microscopy images of ESC colonies expressing reporter genes in suspension. Green arrowheads, EGFP-expressing colony. Magenta arrowheads, mKate2-expressing colony. Scale bar, 400 µm. (b) Reporter gene expression of single ESC colonies isolated with the ESC manipulation device. Scale bar, 100 µm.
